# Costimulatory Molecules and Immune Checkpoints Are Differentially Expressed on Different Subsets of Dendritic Cells

**DOI:** 10.3389/fimmu.2019.01325

**Published:** 2019-06-11

**Authors:** Claudia Carenza, Francesca Calcaterra, Ferdinando Oriolo, Clara Di Vito, Marta Ubezio, Matteo Giovanni Della Porta, Domenico Mavilio, Silvia Della Bella

**Affiliations:** ^1^Department of Medical Biotechnologies and Translational Medicine, University of Milan, Milan, Italy; ^2^Lab of Clinical and Experimental Immunology, Humanitas Clinical and Research Center, Rozzano, Italy; ^3^Cancer Center, Humanitas Reserach Hospital, Rozzano, Italy; ^4^Humanitas University, Rozzano, Italy

**Keywords:** plasmacytoid dendritic cells, conventional cDC1 dendritic cells, conventional cDC2 dendritic cells, PD-L1, ILT2, TIM-3, immune checkpoint, myelodysplastic syndromes

## Abstract

Dendritic cells (DCs) play a crucial role in initiating and shaping immune responses. The effects of DCs on adaptive immune responses depend partly on functional specialization of distinct DC subsets, and partly on the activation state of DCs, which is largely dictated by environmental signals. Fully activated immunostimulatory DCs express high levels of costimulatory molecules, produce pro-inflammatory cytokines, and stimulate T cell proliferation, whereas tolerogenic DCs express low levels of costimulatory molecules, produce immunomodulatory cytokines and impair T cell proliferation. Relevant to the increasing use of immune checkpoint blockade in cancer treatment, signals generated from inhibitory checkpoint molecules on DC surface may also contribute to the inhibitory properties of tolerogenic DCs. Yet, our knowledge on the expression of inhibitory molecules on human DC subsets is fragmentary. Therefore, in this study, we investigated the expression of three immune checkpoints on peripheral blood DC subsets, in basal conditions and upon exposure to pro-inflammatory and anti-inflammatory stimuli, by using a flow cytometric panel that allows a direct comparison of the activatory/inhibitory phenotype of DC-lineage and inflammatory DC subsets. We demonstrated that functionally distinct DC subsets are characterized by differential expression of activatory and inhibitory molecules, and that cDC1s in particular are endowed with a unique immune checkpoint repertoire characterized by high TIM-3 expression, scarce PD-L1 expression and lack of ILT2. Notably, this unique cDC1 repertoire was subverted in a group of patients with myelodysplastic syndromes included in the study. Applied to the characterization of DCs in the tumor microenvironment, this panel has the potential to provide valuable information to be used for investigating the role of DC subsets in cancer, guiding DC-targeting treatments, and possibly identifying predictive biomarkers for clinical response to cancer immunotherapy.

## Introduction

Dendritic cells (DCs) are the single most central players in all immune responses. They are ubiquitous professional antigen-presenting cells that have a crucial role in initiating and shaping immune responses. Human DCs can be subdivided in different subsets that differ from each other in their origin, growth factor requirements, migration patterns, and specialized immunological functions. Under non-inflammatory conditions, bone marrow-derived DC progenitors give rise to developmentally distinct DC subsets that can be identified on a transcriptomic and phenotypic basis and that consist of two subsets of conventional (or myeloid) DCs (cDCs) and one subset of plasmacytoid DCs (pDCs) (1–3). cDCs express myeloid markers including CD11c, and are the most important antigen presenting cells to T lymphocytes. Through the expression of pathogen recognition receptors and cytokine receptors, cDCs can be activated differentially by distinct signals coming from microbes, dying cells and immune cells. Type 1 cDCs (cDC1s) require the transcription factors IRF8, BATF3, and ID2 for their development (4), and express CD141 (BDCA-3) and other markers on their surface (1, 4, 5). They play a crucial role in immune responses against intracellular pathogens and cancer, mainly related to their intrinsic ability to efficiently up-take and cross-present antigens, and to activate T helper 1 (Th1) and cytotoxic T cell responses (4, 6, 7). cDC1s isolated from human lung have also been demonstrated to efficiently activate Th2 polarization (5). Type 2 cDCs (cDC2s) require the transcription factors IRF4, IRF2, and Traf6 for their development (4), and express CD1c (BDCA-1) and other markers on their surface (1, 7). Molecularly equipped to generate Th17 responses (8), cDC2s promote immune responses against extracellular bacteria and fungi (4). Moreover, unlike murine cells, human cDC2s have also been demonstrated to cross-present antigens and produce IL-12 upon proper stimulation, thus sharing with cDC1s the ability to activate cytotoxic and Th1 immune responses in some specific conditions (7, 9, 10). pDCs lack myeloid markers, while express CD123 (IL-3 receptor α-chain) and other pDC-restricted markers, such as CD303 (BDCA-2) and CD304 (BDCA-4). They are unique in their ability to rapidly produce type I IFN upon viral infections, property that confers these cells a primary role in anti-viral defenses (11). Upon opportune stimulation, pDCs can also cross-present antigens and promote other inflammatory responses, supporting a multifaceted function of this DC subset in the activation of adaptive immune responses (9, 11, 12). Upon inflammatory conditions, additional subsets of inflammatory DCs can infiltrate the site of inflammation. Although their ontology is difficult to address in humans, transcriptomic analyses suggest that inflammatory DCs derive from monocytes rather than from DC precursors (7, 13, 14), monocyte differentiation into monocyte-derived DCs (moDCs) being driven by the activation of the aryl hydrocarbon receptor (15). moDCs are present in many different human steady-state tissues, and their number rapidly increases upon inflammation [reviewed in (7)]. Despite great phenotypic heterogeneity among moDCs obtained from different anatomic sites, tissue-resident moDCs usually express CD1a (7, 16, 17). Inflammatory DCs also include a small population of 6-sulfo-LacNAc (slan)-positive cells (2, 7). Although slanDCs circulating in the blood have a transcriptional profile that overlap with the profile of CD16^+^ non-classical monocytes thus suggesting a monocyte origin of these cells (2, 18), slanDCs in peripheral tissues are endowed with DC functions including efficient antigen presentation, ability to activate naive T cells and promote Th1/Th17 immune responses (19, 20).

Notably, despite the existence of a functional specialization of distinct DC subsets, the effects of DCs on adaptive immune responses partly depend on the subset they belong to, and partly depend on the functional state of DCs, which is largely dictated by environmental signals. In particular, depending on the signals received from the microenvironment, DCs can either activate adaptive immune responses or mediate immune tolerance (21). Immunogenic DCs are characterized by high expression of costimulatory molecules, production of pro-inflammatory cytokines, and ability to stimulate T cell proliferation, whereas tolerogenic DCs express low levels of costimulatory molecules, produce immunomodulatory cytokines, and impair T cell proliferation (22). Signals coming from inhibitory receptors expressed on DC surface may also contribute to the tolerogenic behavior of DCs, but our knowledge on this possible pathway in regulating the activity of DCs is scarce at the moment.

The introduction of immune checkpoint inhibitors in cancer immunotherapy has recently revolutionized cancer treatment, providing unprecedented clinical benefits (23). Immune checkpoints are proteins that restrict physiologic immune cell responses in order to maintain immune homeostasis and protect host tissues from unnecessary damage due to excessive inflammation (24). Although at present the efficacy of immune checkpoints inhibitors is well-established in oncology, there is increasing evidence that their use may also be effective in several non-cancer acute and chronic inflammatory conditions, including sepsis, burns, and chronic infections (25). Moreover, innovative strategies aimed at enhancing the signaling of immune checkpoints in autoimmune diseases are under investigation (26). Understanding whether DC function is regulated by signals generated from immune checkpoints expressed on DC surface will help to understand whether the clinical efficacy of immune checkpoint inhibitors may rely to some extent on their effects on DCs. As the knowledge on the expression of immune checkpoints on human DCs is only fragmentary at present, mapping the expression of these inhibitory molecules on DC subsets at rest and upon exposure to different conditions represents the first step in this direction.

Therefore, in this study we investigated the expression of three immune checkpoints, namely programmed death-ligand 1 (PD-L1), immunoglobulin-like transcript 2 (ILT2), and T cell immunoglobulin and mucin domain-3 (TIM-3) on peripheral blood DC subsets, in basal conditions and upon exposure to pro-inflammatory and anti-inflammatory stimuli. To this aim, we developed a flow cytometric panel that allows to identify in a single tube cDC1s, cDC2s, pDCs, moDCs, and slanDCs, and to assess their expression of up to six surface molecules. In order to provide a profile of the activatory/tolerogenic phenotype of DC subsets, we included in our analysis the expression of three main costimulatory molecules, namely CD40, CD80, and CD86. We applied this tube to the analysis of whole blood samples stimulated with the TLR4-ligand lipopolysaccharide (LPS), the TLR7-ligand imiquimod (IMQ) and the immunosuppressive cytokine IL-10, either alone or in combination. After proving the reliability of our assay by confirming some previous observations regarding the expression of costimulatory molecules on peripheral blood DCs, we demonstrated important differences in the expression of immune checkpoints among DC subsets that may bring novel insights into the comprehension of DC heterogeneity and the differential mechanisms used by DC subsets to control immune responses. In order to test the newly developed panel with the characterization of DCs in a pathologic context, we further applied our 18-color method to the study of peripheral blood DCs in patients affected by myelodysplastic syndromes (MDS), a heterogeneous group of haematopoietic neoplasms characterized by ineffective haemopoiesis and progression to acute myeloid leukemia in a third of patients (27). Our results highlighted, indeed, important numerical and immunophenotypic changes of DC subsets in these patients.

## Materials and Methods

### Subjects

Peripheral blood was obtained by venipuncture from 16 healthy volunteers (9 females, 7 males, mean age 39 years, range 24–62) and was anti-coagulated with sodium heparin. Ten patients with MDS whose clinical features are reported in Table 1 were also enrolled. Patients receiving chemotherapy, hypomethylating agents, or luspatercept were excluded. The study protocol was approved by the institutional review boards (IRB) of Humanitas Research Hospital (ONC-OSS-04-2017; 29/18). Written informed consents were provided by all participants before inclusion in the study in compliance with the Declaration of Helsinki.

**Table 1:** Clinical features of MDS patients (*n* = 10).

Age, years (mean, range)	72 (42–92)
Sex, males:females (*n*)	5:5
**Hemoglobin (*n*)**	
<10.0 g/dl	7
≥10.0 g/dl	3
**Neutrophil Count (*n*)**	
<1.8 ×10^9^/L	4
≥1.8 ×10^9^/L	6
**Platelet Count (*n*)**	
<100 ×10^9^/L	4
≥100 ×10^9^/L	6
**Transfusion Dependency (*n*)**	
Yes	5
No	5
**WHO Classification (*n*)**	
AML secondary to MDS	1
MDS-RS-MLD	1
MDS-MLD	2
MDS-RS-SLD	3
MDS-SLD	1
MDS-EB-1	1
MDS-EB-2	1
**Bone Marrow Fibrosis (*n*)**	
Yes	1
No	9
**Bone Marrow Blasts (*n*)**	
<5%	7
≥5 to <10%	1
≥10%	2
**IPSS Risk Score (*n*)**	
Low	6
int-1	2
High	1
**IPSS-R Risk Score (*n*)**	
Very low	1
Low	6
Int	1
Very high	1
**Therapy at Blood Withdrawal**	
Erythropoietin	2

### Stimulation of WB Samples With TLR-Ligands and IL-10

Whole blood (WB) stimulation with TLR-ligands was performed as previously described (28). Briefly, WB samples diluted v/v in RPMI 1640 medium (Euroclone, Wetherby, West York, UK) were incubated at 37°C in a 5% CO_2_ humidified atmosphere for 5 h in the absence or presence of either LPS (serotype 055:B5; 100 ng/ml; Sigma Chemicals Co.) or IMQ (10 μg/ml; InvivoGen, San Diego, CA), both prepared according to the manufacturer's recommendations. The concentrations of LPS and IMQ as well as the duration of cultures were established based on previous studies (29–34). Incubation with IL-10 was performed by adding IL-10 (recombinant human IL-10; 40 ng/ml; Peprotech, London, UK) either alone or in combination with LPS or IMQ.

### Sample Staining

At the end of the culture, WB samples (500 μl) were stained with an 18-color flow cytometry panel of monoclonal antibodies (mAbs) as described below. Briefly, samples were incubated with ammonium chloride for 10 min to lyse erythrocytes. After washing, they were stained with the Fixable Viability Stain 780 (BD Biosciences), then washed and stained with the combination of mAbs listed in Table 2. mAbs specific for lineage markers, HLA-DR, CD123, CD11c, CD1c, CD141, CD1a, M-DC8 (anti-slan) were used to gate on DC subsets, while the other mAbs were used to assess the immunostimulatory/regulatory phenotype of each DC subset. Staining conditions for each mAb were preliminarily determined in titration assays, as previously described (35). All operations were done at 4°C in the dark.

**Table 2:** List of monoclonal antibodies used in this study.

**Marker**	**Description**	**Clone**	**Conjugate**	**Manufacturer**	**Batch**	Titer (μl)^*^
CD45	Leukocyte common antigen	HI30	AF700	BD Biosciences	8186553	1.25
CD3	Lineage marker—T cells	HIT3A	FITC	Biolegend	B218086	1.25
CD19	Lineage marker—B cells	4G7	FITC	BD Biosciences	8162764	2.5
CD20	Lineage marker—B cells	2H7	FITC	BD Biosciences	3291707	2.5
CD56	Lineage marker—NK cells	NCAM 16.2	FITC	BD Biosciences	61126	0.3
CD14	Lineage marker—monocytes	M5E2	BV570	Biolegend	B225361	1.25
CD16	Lineage marker—NK cells and granulocytes	3G8	BUV496	BD Biosciences	8116651	0.6
HLA-DR	Major histocompatibility complex class II molecule	G46-6	BUV661	BD Biosciences	7249926	0.15
CD123	pDC marker	7G3	PE-Cy7	BD Biosciences	8060955	0.6
CD11c	mDC marker	B-ly6	PE-Cy5	BD Biosciences	80859	1.25
CD141 (BDCA-3)	cDC1 marker	M80	BV605	Biolegend	B239279	1.25
CD1c (BDCA-1)	cDC2 marker	L161	BV421	Biolegend	B227045	1.25
M-DC8 (anti-slan)	slanDC marker	DD-1	APC	Milteny Biotec	5180403606	5
CD1a	moDC marker	HI149	BUV395	BD Biosciences	7227951	0.6
CD40	Costimulatory molecule	5C3	BV650	BD Biosciences	8163659	0.6
CD80	Costimulatory molecule	L307	BV510	BD Biosciences	8228546	0.3
CD86	Costimulatory molecule	2331	BUV737	BD Biosciences	7240739	0.6
CD274 (PD-L1)	Inhibitory molecule	MIH1	PE-CF594	BD Biosciences	7191550	2.5
CD85j (ILT2)	Inhibitory molecule	GHI/75	PE	Biolegend	B222938	2.5
CD366 (TIM-3)	Inhibitory molecule	7D3	BV711	BD Biosciences	7348783	0.3
Fixable Viability Stain 780	Viability marker		APC-Cy7	BD Biosciences	6174894	0.025

* *staining in 100 μl*.

### Flow Cytometry Data Acquisition and Analysis

**Figure 1:**
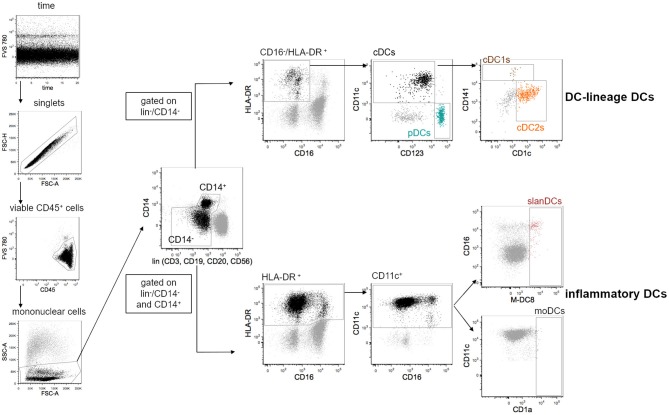
Gating strategy used for the identification of 5 distinct DC subsets in the peripheral blood of healthy donors. DCs were analyzed within the gate of single viable mononuclear cells. Lineage-DCs were identified in the gate of lin^−^/CD14^−^/CD16^−^/HLA-DR^+^ cells. Within lineage-DCs, pDCs (CD123^+^/CD11c^−^), cDC1s (CD123^−^/CD11c^+^/CD141^+^) and cDC2s (CD123^−^/CD11c^+^/CD1c^+^) were identified. Inflammatory DCs were identified as lin^−^/HLA-DR^+^/CD11c^+^ that could be negative or positive for CD14 and CD16. Within inflammatory DCs, slanDCs, and moDCs were identified based on positive staining of M-DC8 and CD1a, respectively. As expected, moDCs were undetectable in most samples.

All data were acquired on a FACS Symphony A5 flow cytometer (BD Biosciences) equipped with five lasers (UV, 350 nm; violet, 405 nm; blue, 488 nm; yellow/green, 561 nm; red, 640 nm; all tuned at 100 mW, except UV tuned at 60 mW). Flow Cytometry Standard (FCS) 3.0 files were imported into FlowJo software version 9.9.6 (FlowJo LLC, Ashland, Oregon), and data were compensated by using single-stained antibody-capture beads (CompBeads, BD Biosciences), as previously described (36). Flow cytometry data were analyzed by standard gating to remove aggregates and dead cells. The gating strategy used to define DC subsets is shown in Figure 1. Because peripheral blood DCs are characterized by forward scatter (FSc) similar to monocytes and side scatter (SSc) similar to lymphocytes, an acquisition gate was established based on FSc and SSc that included both lymphocytes and monocytes (mononuclear cells) but excluded most granulocytes and debris. DC-lineage DCs were defined as cells negative for lineage markers (lin: CD3, CD19, CD20, CD56), CD14 and CD16, and positive for HLA-DR expression. Gated on these cells, pDCs and cDCs were identified based on the expression of CD123 and CD11c, respectively. Within cDCs, cDC1s, and cDC2s were further identified based on the expression of CD141 and CD1c, respectively. Because inflammatory DCs can express CD14 and CD16, these two markers were considered separately from the pool of the other lineage markers, and inflammatory DCs were identified gated on cells that were negative for CD3, CD19, CD20, and CD56, but that could be negative or positive for CD14 and CD16. Inflammatory DCs were further identified as being positive for the expression of HLA-DR and CD11c. Gated on these cells, slanDCs were identified based on M-DC8 expression, moDCs based on CD1a expression. The expression of the costimulatory molecules CD40, CD80 and CD86, and the inhibitory molecules PD-L1, ILT2, and TIM-3 was analyzed gated on each DC subset. Fluorescence Minus One (FMO) controls for each of these molecules were performed. Data were expressed as net mean fluorescence intensity (MFI), calculated by subtracting the respective FMO's MFI from the sample's MFI (37).

### tSNE-Based Unsupervised Analysis

t-distributed stochastic neighbor embedding (tSNE) analysis was performed as previously described (38). In particular, a unique computational barcode was assigned to single samples. For each sample, events gated on live CD45^+^/lin^−^/HLA-DR^+^ cells were subsequently concatenated and visualized with tSNE (Barnes-Hut implementation; iterations, 800; perplexity, 20; initialization, deterministic; theta, 0.5; eta: 200). The expression of the following markers was considered: CD14, CD16, HLA-DR, CD11c, CD123, CD141, CD1c, M-DC8, CD1a, CD40, CD80, CD86, PD-L1, ILT2, TIM-3.

### Statistical Analysis

Data were shown as mean ± standard error of the mean. Paired *t*-test was used for comparison between DC subsets, and to assess the effects of treatment within each subset. Unpaired *t*-test was used for comparison between MDS patients and healthy donors. All statistical analyses assumed a two-sided significance level of 0.05. Statistical analyses were performed using GraphPad Prism version 7 (GraphPad Software, La Jolla, CA).

## Results

### The New 18-Color Flow Cytometric Method Allows the Identification of DC-Lineage and Inflammatory DC Subsets in Healthy Donors at Expected Rates

By applying our new DC-dedicated 18-color panel to the analysis of DC subsets in whole blood samples obtained from healthy donors, we observed that the frequency of pDCs in mononuclear cells was 0.44 ± 0.06 and the frequency of total DC-lineage cDCs was 0.79 ± 0.08. Further distinguishing between cDC subsets, we observed that the frequency of cDC1s and cDC2s in mononuclear cells were 0.02 ± 0.005 and 0.49 ± 0.03, respectively. Within inflammatory DCs, the frequency of slanDCs in mononuclear cells was 0.07 ± 0.03, while moDCs were almost undetectable in all WB samples, as expected. All together, the frequencies of all DC subsets in the mononuclear cell population were similar to values reported in previous studies by us and other Authors (10, 20, 29, 30, 32, 39–41), thus indicating that the newly developed 18-color panel allowed a reliable identification of the investigated DC subsets.

### Expression of Costimulatory and Inhibitory Molecules on DC Subsets From Healthy Donors in Basal Conditions

**Figure 2:**
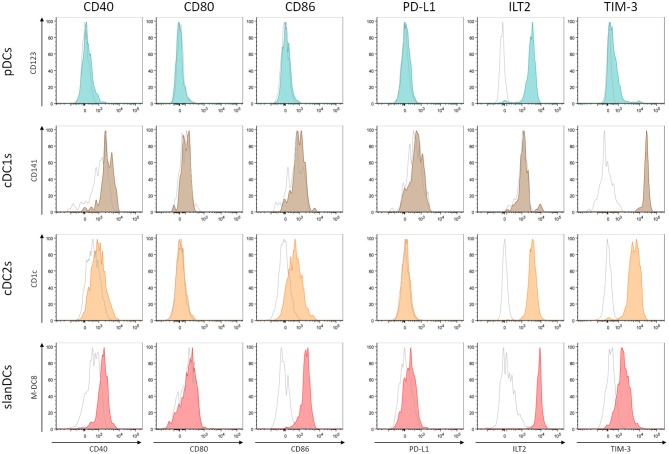
Analysis of costimulatory and inhibitory molecule expression on DC subsets. The expression of 3 costimulatory molecules (CD40, CD80, and CD86) and 3 inhibitory molecules (PD-L1, ILT2, and TIM-3) was analyzed on pDCs (shown in dark turquoise), cDC1s (brown), cDC2s (orange), slanDCs (red). Each graph shows the overlay of the stained sample (colored) and the appropriate FMO control (shown in empty gray). A representative analysis is shown.

**Figure 3:**
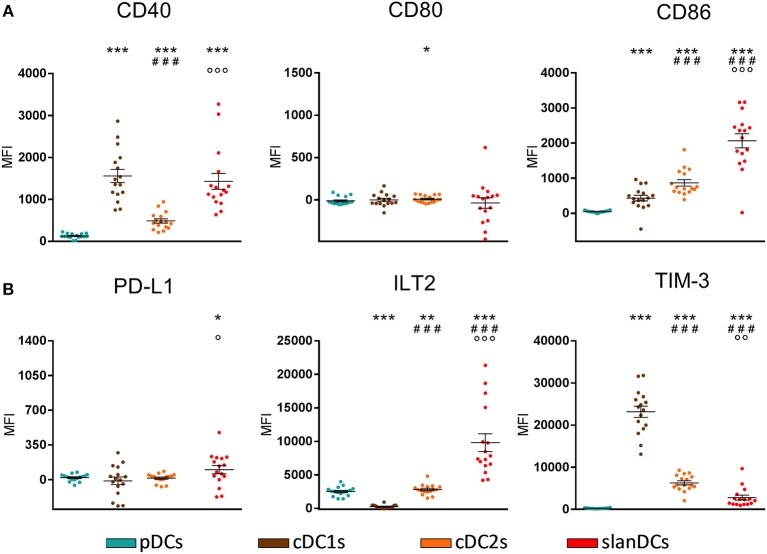
Surface expression of costimulatory and inhibitory molecules on DC subsets in basal conditions. The expression levels of the costimulatory molecules CD40, CD80, CD86 **(A)** and the inhibitory molecules PD-L1, ILT2, TIM-3 **(B)** were assessed on pDCs, cDC1s, cDC2s, and slanDCs, and expressed as net MFI. Data were obtained from 16 healthy donors. Each symbol represents a single sample. In each series, the mean ± SEM is also shown. *p < 0.05, ^**^p < 0.01, ^***^p < 0.001 vs. pDCs. ^###^*p* < 0.001 vs. cDC1s. ^◦^*p* < 0.05, ^◦◦^*p* < 0.01, ^◦◦◦^*p* < 0.001 vs. cDC2s. Statistical significance calculated using the paired *t*-test.

The activation state of each DC subset in unstimulated conditions was assessed as the surface expression of the costimulatory molecules CD40, CD80, and CD86; their potentially regulatory function as the surface expression of the inhibitory molecules PD-L1, ILT2, and TIM-3. The intensity of expression of each of these molecules was simultaneously analyzed in the gate of pDCs, cDC1s, cDC2s, and slanDCs in the same tube, thus allowing a direct comparison between subsets. The expression on moDCs was not considered, as this subset was undetectable in WB samples. A representative analysis is shown in Figure 2. By using this strategy, we observed that the expression of the costimulatory molecules, expressed as net MFI, was quite variable among DC subsets, as shown in Figure 3A. According to previous reports, CD80 expression in basal conditions was negligible in all subsets, and the expression of CD40 and CD86 was lower on pDCs than on cDC subsets (36, 42). Besides these expected results, by directly comparing cDC1s and cDC2s, our immunophenotypic analysis indicated that cDC1s expressed higher levels of CD40 and lower levels of CD86 than cDC2s. Moreover, according to their inflammatory function, slanDCs expressed higher CD40 and CD86 levels than cDC2s. As shown in Figure 3B, also the expression of the inhibitory molecules PD-L1, ILT2, and TIM-3 was quite variable among DC subsets. PD-L1 was expressed at low levels on slanDCs and was negligible in all other subsets. ILT2 was expressed in basal conditions on pDCs and cDC2s, its expression was higher on slanDCs, and negligible on cDC1s. On the contrary, TIM-3 was expressed at the highest levels on cDC1s.

### Expression of Costimulatory Molecules on DC Subsets From Healthy Donors Upon Exposure to Pro-inflammatory and Anti-inflammatory Stimuli

**Figure 4:**
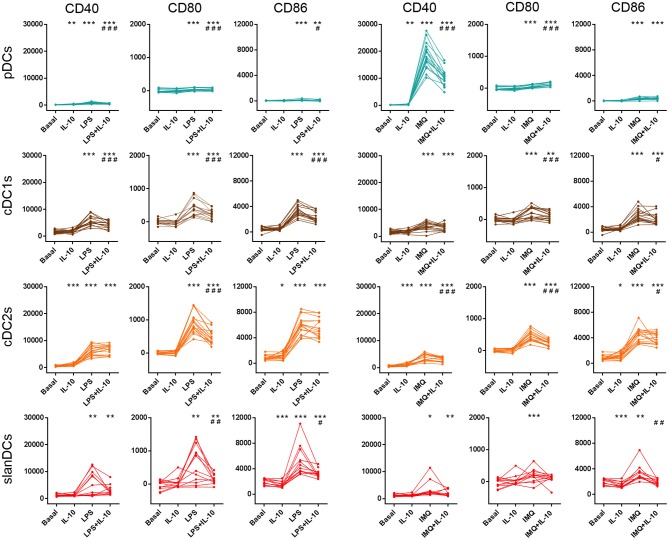
Surface expression of costimulatory molecules on DC subsets upon exposure to pro-inflammatory and anti-inflammatory stimuli. The expression of CD40, CD80, and CD86 was assessed on DCs after 5-h incubation of whole blood samples in the absence or presence of LPS, IMQ, and IL-10 either alone or in combination. The expression level of each costimulatory molecule was expressed as net MFI. Color legend for DC subsets as reported in legend to Figure 3. Data were obtained from 16 healthy donors. ^*^p < 0.05, ^**^p < 0.01, ^***^p < 0.001 vs. basal. #*p* < 0.05, ^##^*p* < 0.01, ^###^*p* < 0.001 vs. cells treated with TLR-ligand only. Statistical significance calculated using the paired *t*-test.

The ability of DC subsets to undergo TLR-induced activation was assessed upon exposure to LPS and IMQ, as they had been previously identified as the best TLR-ligands to be used in WB assay (28). The activation state of pDCs, cDC1s, cDC2s, and slanDCs was assessed as cell surface expression of the costimulatory molecules CD40, CD80, and CD86, using the same strategy described for the immunophenotypic characterization of DC subsets in unstimulated conditions. In order to challenge DC subsets with pro-inflammatory and anti-inflammatory stimuli, WB samples were incubated in the absence or presence of LPS, IMQ and IL-10, either alone or in combination. As shown in Figure 4, stimulation of WB samples with the TLR4-ligand LPS induced full activation of both cDC subsets and slanDCs, and only marginal activation of pDCs. Stimulation of WB samples with the TLR7-ligand IMQ induced the highest CD40 upregulation in pDCs, but also activated cDC subsets and slanDCs. This lack of selectivity of TLR ligands on either DC subset when stimulating WB samples is in accordance with our previous observations, and likely mediated by indirect cytokine-mediated effects related to the presence of mixed populations (28). Notably, upon stimulation with either LPS or IMQ, a higher upregulation of CD80 and CD86 was observed in cDC2s than cDC1s (*p* < 0.001 in all cases). Moreover, the upregulation of costimulatory molecules induced by LPS and IMQ was partially reverted by the anti-inflammatory cytokine IL-10, that scarcely affected the expression of costimulatory molecules on any DC subsets when administered alone.

### Expression of Inhibitory Molecules on DC Subsets From Healthy Donors Upon Exposure to Pro-Inflammatory and Anti-inflammatory Stimuli

**Figure 5:**
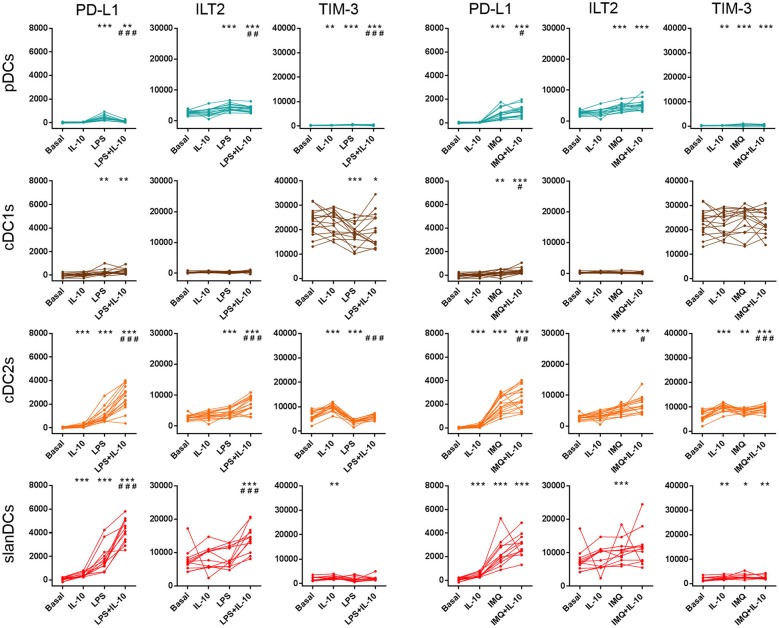
Surface expression of immune checkpoints on DC subsets upon exposure to pro-inflammatory and anti-inflammatory stimuli. The expression of PD-L1, ILT2, and TIM-3 was assessed on DCs after 5-h incubation of whole blood samples in the absence or presence of LPS, IMQ, and IL-10 either alone or in combination. The expression level of each inhibitory molecule was expressed as net MFI. Color legend for DC subsets as reported in legend to Figure 3. Data were obtained from 16 healthy donors. ^*^p < 0.05, ^**^p < 0.01, ^***^p < 0.001 vs. basal. ^#^*p* < 0.05, ^##^*p* < 0.01, ^###^*p* < 0.001 vs. cells treated with TLR-ligand only. Statistical significance calculated using the paired *t*-test.

In order to investigate the ability of the above mentioned DC subsets to change their surface expression of inhibitory molecules upon exposure to pro-inflammatory and anti-inflammatory conditions, in the same samples incubated with LPS, IMQ and IL-10, and analyzed for the expression of costimulatory molecules, we also analyzed the expression of the inhibitory molecules PD-L1, ILT2, and TIM-3. As shown in Figure 5, incubation of WB samples with TLR-ligands induced an overall upregulation of the immune checkpoints PD-L1 and ILT2 on DCs, suggesting that upregulation of these inhibitory molecules during DC stimulation may represent a mechanism aimed at preventing the activation of excessive immune responses. According to the loss of selectivity of TLR-ligands during WB stimulation, both LPS and IMQ affected the expression of inhibitory molecules on pDCs and cDCs, although to a different extent. Within cDC subsets, exposure to TLR-ligands induced a higher upregulation of PD-L1 on cDC2s than cDC1s (*p* < 0.0001 in both cases), and increased the expression of ILT2 on cDC2s only. Consistent with its immunosuppressive role, IL-10 added during LPS or IMQ stimulation further increased PD-L1 and ILT2 expression in most cases, though it was scarcely effective when administered alone. Finally, the expression of TIM-3 on DC subsets was poorly affected by WB exposure to TLR-ligands and/or IL-10, as it maintained its highest expression on cDC1s, a lower expression on cDC2s and slanDCs, and its lowest expression on pDCs, whatever the stimulatory conditions were.

### Characterization of Peripheral Blood DC Alterations in MDS Patients by Applying the New Flow Cytometric Panel

**Figure 6:**
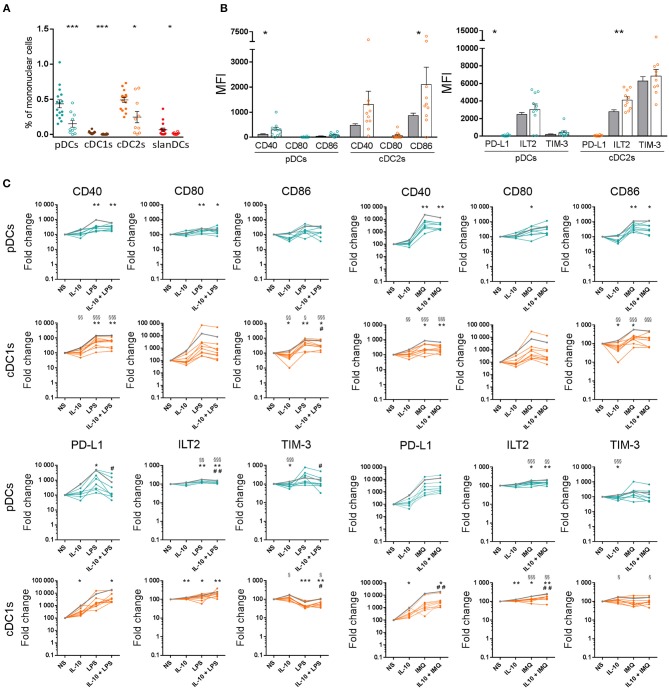
Characterization of peripheral blood DC alterations in MDS patients. **(A)** The frequency of DC subsets in whole blood samples obtained from healthy donors (full circles, *n* = 16) and MDS patients (empty circles, *n* = 10), was expressed as percentage of mononuclear cells. **(B)** The expression levels of the costimulatory molecules CD40, CD80, CD86 and the inhibitory molecules PD-L1, ILT2, TIM-3 were assessed on pDCs and cDC2s of healthy donors (gray bars) and MDS patients (white bars behind colored empty circles) and expressed as net MFI. In each series, the mean ± SEM is also shown. ^*^p < 0.05, ^**^p < 0.01, ^***^p < 0.001 vs. healthy controls. Statistical significance calculated using the *t*-test. **(C)** Surface expression of costimulatory molecules and immune checkpoints on pDCs and cDC2s upon exposure to pro-inflammatory and anti-inflammatory stimuli in healthy controls (mean shown as gray line, *n* = 16) and MDS patients (colored lines, each line corresponding to one patient, *n* = 10). The expression of CD40, CD80, CD86, PD-L1, ILT2, and TIM-3 was assessed on DCs after 5-h incubation of whole blood samples in the absence or presence of LPS, IMQ, and IL-10 either alone or in combination. The expression level of each analyzed molecule was expressed as fold change of the net MFI normalized on untreated sample (Basal). ^§^*p* < 0.05, ^§§^*p* < 0.01, ^§§§^*p* < 0.001 MDS vs. healthy controls. Statistical significance calculated using the *t*-test. ^*^p < 0.05, ^**^p < 0.01, ^***^p < 0.001 vs. MDS basal. ^#^*p* < 0.05, ^##^*p* < 0.01 vs. MDS cells treated with TLR-ligand only. Statistical significance calculated using the paired *t*-test.

In order to test the newly developed panel on the characterization of DCs in a pathologic context, we applied our 18-color method to the study of peripheral blood DCs in patients with MDS, and compared the results with DC features observed in healthy donors. As shown in Figure 6A, all circulating DC subsets, namely pDCs, cDC1s, cDC2s, and slanDCs, were significantly reduced in the blood of MDS patients compared with controls. The reduction of DC subsets was not correlated with disease severity, as the frequency of DC subsets did not differ between patients with low IPSS or IPSS-R risk score, and patients with higher scores. Because of DC reduction, the analysis of costimulatory and checkpoint molecule expression on cDC1s and slanDCs in MDS patients was hampered by the low number of cells. Therefore, the immunophenotypic characterization in these patients was performed only on pDCs and cDC2s. As shown in Figure 6B, in unstimulated conditions both subsets tended to be more activated, with a significantly higher expression of CD86 on MDS than control cDC2s. The same cells also showed a higher expression of the inhibitory molecule ILT2. We further investigated the ability of DCs from MDS patients to change their surface expression of costimulatory and inhibitory molecules upon exposure to LPS, IMQ and IL-10, alone or in combination. As shown in Figure 6C, similar to control DCs, both pDCs and cDC2s underwent upregulation of CD40, CD80, and CD86 upon stimulation with LPS and IMQ that was partially reverted by addition of IL-10. Notably, TLR-induced fold change in costimulatory molecule expression was significantly lower in MDS than control DCs, indicating that DCs from MDS patients were hyporesponsive to TLR stimulation. As shown in the same figure, similar results were observed when the expression of inhibitory checkpoints was analyzed. No correlation was observed between the grade of TLR hyporesponsiveness and disease severity.

### tSNE Analysis Reveals Changes in cDC1s From MDS Patients and Heterogeneity of cDC2s Upon TLR-Mediated Stimulation

**Figure 7:**
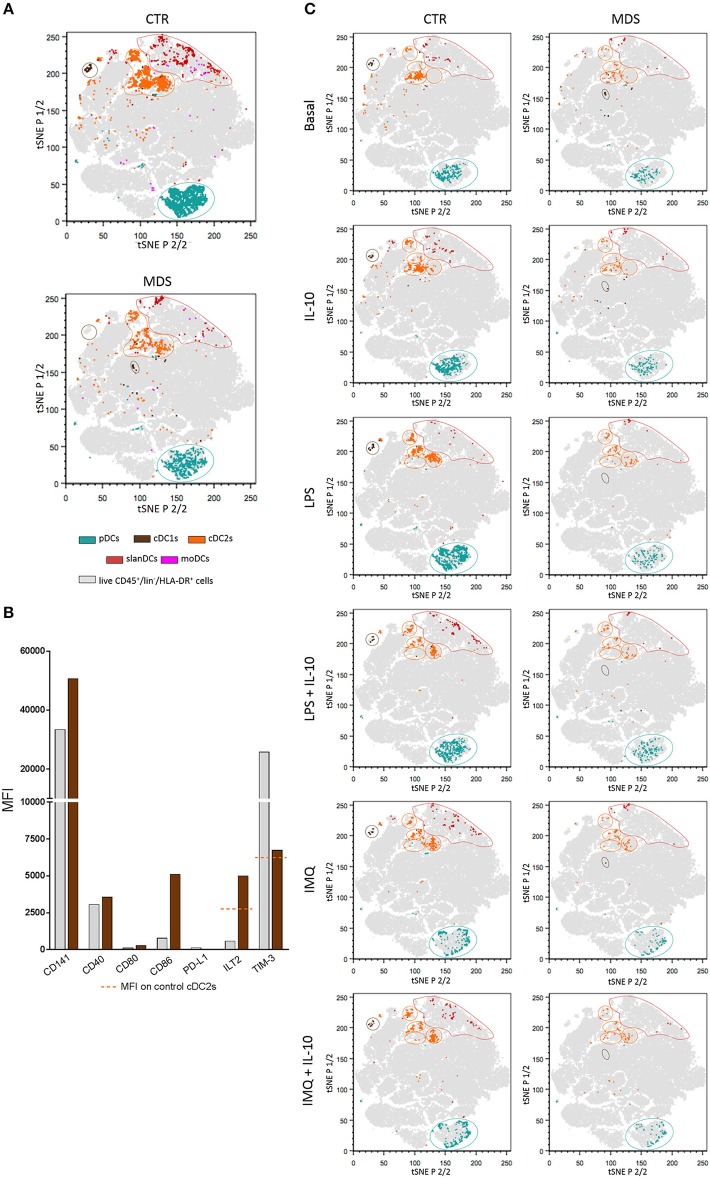
tSNE plots showing the clustering of peripheral blood DC subsets in healthy donors and MDS patients. Live CD45^+^/lin^−^/HLA-DR^+^ cells obtained from all unstimulated and stimulated samples of healthy donors (*n* = 16) and MDS patients (*n* = 10) were concatenated and displayed in a single tSNE dot plot (shown in gray). **(A)** tSNE plots showing clustered DC subsets in pooled unstimulated and stimulated samples of healthy controls (upper plot) compared with MDS patients (lower plot). Color code as indicated. Note the different geographic location of cDC1s in patients and controls. **(B)** The expression of CD141, costimulatory molecules (CD40, CD80, CD86), and immune checkpoints (PD-L1, ILT2, and TIM-3) was assessed on cDC1s from healthy donors (gray bars) and MDS patients (brown bars). Data expressed as MFI measured on concatenated files from all unstimulated samples. **(C)** tSNE plots showing clustered DC subsets in each single culture condition, in controls (left column) and MDS patients (right column). Note the different geographical location of unstimulated and TLR-stimulated cDC2s.

In order to perform a multidimensional analysis, we further analyzed our flow cytometric data by using unsupervised tSNE algorithm. Live CD45^+^/lin^−^/HLA-DR^+^ cells obtained from all unstimulated and stimulated samples of healthy donors and MDS patients were concatenated and displayed in a single tSNE dot plot. Figure 7A shows DC subsets obtained from all unstimulated and stimulated samples of 16 healthy donors compared with 10 MDS patients. As shown in the Figure, pDCs (shown in dark turquoise), cDC2s (orange), and slanDCs (red) obtained from patients fell in the same tSNE regions as healthy donors. On the contrary, cDC1s (brown) of MDS patients fell in a tSNE region different from that of control cDC1s, suggesting the existence of profound differences between these two cell populations. In order to investigate this issue, we compared the immunophenotype of cDC1s obtained from patients and controls. Because of the severe reduction of this cell subset in the patients' blood, the analysis was performed on concatenated files of all unstimulated samples. As shown in Figure 7B, cDC1s of MDS patients were characterized by higher expression of CD141 and CD86 than healthy donors. Notably, cDC1s of MDS patients also showed a huge upregulation of ILT2 expression to levels even higher than control cDC2s, and a downregulation of TIM-3 expression to levels similar to control cDC2s, indicating an overall subversion of the immune checkpoint repertoire of cDC1s in MDS patients. Finally, in order to assess the impact of stimulation on DC geographical location in tSNE plots, we compared tSNE plots showing DC subsets obtained in each single culture condition. As shown in Figure 7C, unlike the other DC subsets that maintained the same position in the tSNE plot regardless of the culture conditions, upon stimulation cDC2s clusterized into three different regions that did not overlap with the region of unstimulated cDC2s. This finding may suggest that, upon TLR-induced stimulation, cDC2s underwent more profound changes compared with the other DC subsets analyzed. However, the comparison of concatenated files obtained from the three clusters of stimulated cDC2s failed to highlight substantial differences among clusters; the comparison of these clusters with the cluster of unstimulated cDC2s did not demonstrate additional differences between unstimulated and stimulated clusters, beyond the obvious parameters already described by the analysis of each single tube.

## Discussion

In this study, we investigated the differential expression of major activatory and inhibitory molecules on peripheral blood DC subsets, in basal conditions and upon *in vitro* exposure to inflammatory and anti-inflammatory stimuli. To this aim, we developed a 18-color flow cytometry panel dedicated to the immunophenotypic characterization of DC subsets. By applying this panel to the analysis of whole blood samples obtained from healthy donors, on the one hand we could confirm some previous observations, thus providing evidence for assay reliability; on the other hand, we observed important differences in the expression of activatory and inhibitory molecules among DC subsets, that may bring novel insights into the comprehension of DC heterogeneity.

In particular, by directly comparing the two cDC subsets, we observed that in basal conditions cDC1s are characterized by higher CD40 and lower CD86 expression than cDC2s. Similar results were reported in a previous study investigating the expression of costimulatory molecules on cDC subsets (42), but were not confirmed in other studies (39, 43, 44). Although we do not have evident explanations for these discrepancies, our observation that the difference in CD86 expression was maintained upon TLR-mediated stimulation, together with the knowledge that CD40 and CD86 play different roles in immune activation (45, 46), may strengthen the relevance of our finding and suggest a potential contribution of these molecules to the functional specialization of DC subsets that may deserve further investigation in future studies.

Moreover, in this study we observed a great variability among DC subsets in the expression of the checkpoint molecules PD-L1, ILT2, and TIM-3. This observation may be particularly relevant in view of the increasing interest in understanding the role played by DCs during immune checkpoint blockade in cancer immunotherapy. In fact, remarkable and long-term responses to these treatments are achieved only in a proportion of patients (23). Understanding the reasons for patient variability in response to therapy and developing reliable biomarkers to predict patients who are likely to respond to checkpoint inhibitors remains a challenge. In the case of PD-1/PD-L1 blockade, PD-L1 is a ligand of the co-inhibitory receptor programmed death-1 (PD-1) that is expressed on activated T cells and that, if engaged by its ligands, suppresses T cell function primarily by inactivating CD28 signaling (47). Because many tumors express PD-L1, the rationale of PD-L1 pathway blockade is to inhibit the immunosuppressive PD-L1/PD-1 interaction between tumor cells and T cells that hampers the activity of CD4^+^ and CD8^+^ T cells (48). Accordingly, the expression of PD-L1 on tumor cells has been used as a predictive biomarker of treatment response in many studies [reviewed in (24)]. However, positive responses to PD-1/PD-L1 blockade are often observed in patients whose tumor cells lack PD-L1 expression, suggesting that additional mechanisms may contribute to treatment efficacy (48). Indeed, a positive response to anti-PD-L1 therapy has been associated with high expression of PD-L1 on tumor-infiltrating immune cells across multiple cancer types, indicating a role for PD-L1^+^ immune cells in suppressing anti-tumor responses, which are re-invigorated on blockade of PD-L1 signaling (49). PD-L1^+^ DCs may contribute to the immunosuppressive tumor microenvironment not only by suppressing the activity of T cells, as demonstrated in many preclinical models [reviewed in (48, 50)], but also by promoting the expansion of regulatory T cells (51) and establishing immunoregulatory interactions with NK cells and other immune cells (50). According to DC heterogeneity, it is possible that the regulation of PD-L1 expression and the effects of PD-L1^+^ DCs may differ among different DC subsets. Indeed, the results of our study seem to support this hypothesis. In fact, consistent with previous studies investigating PD-L1 expression in different experimental models (52–55), we observed that PD-L1 was almost undetectable on all subsets of DC-lineage DCs in basal conditions, and it was significantly upregulated on all subsets upon TLR-driven stimulation. However, the levels of PD-L1 induced by incubation with LPS and IMQ were quite variable among DC subsets, with the highest levels observed on slanDCs and the lowest levels on cDC1s. The *in vivo* relevance of this finding will deserve further investigation, by analyzing PD-L1 expression on the different DC subsets at the tissue level. In particular, relevant to the use of PD-1/PD-L1 inhibitors in cancer immunoherapy, it will be interesting to verify the differential expression of PD-L1 on DC subsets in the tumor microenvironment, which may contribute to PD-L1 upregulation on DCs through the action of various factors including IL-10, as used in our study, and tumor growth factor (TGF)-β (56).

Another interesting result of our study was the demonstration that cDC1s failed to express ILT2, both in unstimulated and TLR-stimulated conditions, while all the other DC subsets showed a basal expression of ILT2 that was further upregulated upon exposure to LPS and IMQ. ILT2 is another important checkpoint, expressed on the surface of various immune cell types. It binds classical and non-classical MHC class I molecules with a higher affinity for HLA-G (57) and, upon interaction with its ligands, it inhibits cell function through ITIM signaling (58). The relevance of the inhibitory role of ILT2 in DCs is supported by the demonstration that ILT2 is upregulated on human tolerogenic DCs differentiated *in vitro* using different agents (59, 60), and it is diminished on peripheral blood DCs of patients with autoimmune diseases (61). As the regulation of ILT2 expression on DCs has been poorly investigated so far, our observation that ILT2 levels are increased on most DC subsets upon TLR-stimulation is interesting and may suggest that, similarly to PD-L1, ILT2 is upregulated on DCs upon pathogen-driven stimulation in order to prevent excessive T cell activation and avoid uncontrolled inflammation. Notably, the complete lack of ILT2 expression on cDC1s, together with the lower PD-L1 expression on these cells, may suggest that cDC1s are regulated by different checkpoints compared with the other DC subsets.

Indeed, we observed that, compared with the other DC populations, cDC1s expressed remarkably higher levels of TIM-3, an immune checkpoint that upon interaction with the nuclear protein HMGB1 limits the release of pro-inflammatory cytokines by DCs, thus weakening type I responses (62). Interestingly, consistent with the superior ability of cDC1s to cross-present antigens and activate cytotoxic T cell responses, TIM-3 also binds phosphatidylserine and through this interaction it favors DC capture of apoptotic cells and cross-presentation (63), thus suggesting that the higher expression of TIM-3 on cDC1s may be coeherent with the functional specialization of this DC subset. Notably, a high expression of TIM-3 on the murine equivalent of human cDC1s has been recently described (64), and an important role of TIM-3 expression on these cells has been suggested by the observation that, in a murine model of breast cancer, treatment with TIM-3-blocking antibody increased the immune-mediated response to therapy through cDC1-dependent, not yet clarified mechanisms (64). In addition, TIM-3 expression is increased on tumor-associated DCs in various human cancers (62, 64). The emerging scenario may be, therefore, that cDC1s, though necessary for anti-cancer immunity, are tamed by the immunosuppressive tumor microenvironment through TIM-3 upregulation, that in turn regulates DC function. In this respect, it is worthy noting that in our study IL-10 alone was insufficient to upregulate TIM-3 expression, suggesting that multiple components of the tumor microenvironment are needed for taming DCs with TIM-3.

Indeed, the results obtained in this study by analyzing peripheral blood DCs in MDS patients suggest the possibility of another, unforeseen, scenario. In fact, we observed a significant decrease of all DC subsets, and this finding is in line with previous studies reporting a reduction of cDCs and pDCs in the blood and bone marrow of MDS patients (65, 66). We also observed an overall activation of cDC2s, and a hyporesponsiveness of pDCs and cDC2s to TLR-mediated stimulation. These findings may likely reflect the chronic hyperactivation of TLRs occurring in MDS (67), and the subsequent desensitization of DCs to further TLR-induced stimulation (68, 69). But the most striking result of our study was the observation that cDC1s from MDS patients undergo profound changes, resulting in their falling in a different tSNE region compared with cDC1s from healthy donors. Relevant to the above considerations on immune checkpoints, cDC1s from MDS patients showed huge upregulation of ILT2 and downregulation of TIM-3 expression, thus denoting an overall subversion of their inhibitory molecule repertoire. Further studies will be needed in order to investigate whether these cDC1 changes are also present in the bone marrow of MDS patients, whether they are sustained by the neoplastic process or they play a causative role in it, whether they are related to the immune dysregulation occurring in MDS (67), or whether they are shared with other pathologic conditions where DCs play a crucial role, including other types of cancer, infections, chronic inflammatory diseases. Further studies on sorted DC subsets will also be needed in order to investigate the molecular mechanisms underlying these cDC1 changes, and the impact of these changes on DC function.

In conclusion, in this study we developed a flow cytometry panel that allows a direct comparison of the activatory/regulatory phenotype of DC-lineage and inflammatory DC subsets. By applying this panel to the study of DCs in the peripheral blood, we demonstrated that functionally distinct DC subsets are characterized by differential expression of activatory and inhibitory molecules, and that cDC1s in particular are endowed with a unique immune checkpoint repertoire characterized by high expression of TIM-3 and scarce or null expression of PD-L1 and ILT2, respectively, suggesting that differential mechanisms are used by different DC subsets to control immune responses. The observation that the immune checkpoint repertoire of cDC1s is subverted in MDS patients may pave the way for understanding the impact of these molecules on DC function. Applied to the study of DCs in the tumor microenvironment, this panel has the potential to provide valuable information to be used for improving our comprehension of the role of distinct DC subsets in cancer, guiding DC-targeting immunotherapy, and possibly identifying predictive biomarkers for clinical response in cancer patients undergoing immune checkpoint blockade treatment.

## Ethics Statement

This study was carried out in accordance with the recommendations of the Institutional Review Boards (IRB) of Humanitas Research Hospital with written informed consent from all subjects. All subjects gave written informed consent in accordance with the Declaration of Helsinki. The study protocol was approved by the institutional review boards (IRB) of Humanitas Research Hospital (ONC-OSS-04-2017; 29/18).

## Author Contributions

CC and FC performed experiments, analyzed data, and wrote the manuscript. FO performed experiments. CDV analyzed data (tSNE analysis). MU and MGDP recruited and selected MDS patients and analyzed data. DM designed research and critically revised the manuscript. SDB designed research, interpreted the data, wrote and revised the manuscript.

### Conflict of Interest Statement

The authors declare that the research was conducted in the absence of any commercial or financial relationships that could be construed as a potential conflict of interest.
